# Hiatus hernia repair with a new-generation biosynthetic mesh: a 4-year single-center experience

**DOI:** 10.1007/s00464-023-10005-0

**Published:** 2023-03-28

**Authors:** Kristjan Ukegjini, Diana Vetter, Valerian Dirr, Christian A. Gutschow

**Affiliations:** 1 Department of Visceral and Transplant Surgery, University Hospital Zurich, Zurich, Switzerland; 2 Department of General, Visceral, Endocrine and Transplant Surgery, Kantonsspital St. Gallen, St. Gallen, Switzerland

**Keywords:** Hiatus hernia, Hiatal hernia, Absorbable mesh, Hiatoplasty, Resorbable mesh, Antireflux surgery

## Abstract

**Background:**

Mesh augmentation is a highly controversial adjunct of hiatus hernia (HH) surgery. The current scientific evidence remains unclear and even experts disagree on indications and surgical techniques. With an aim to avoid the downsides of both non-resorbable synthetic and biological materials, biosynthetic long-term resorbable meshes (BSM) have recently been developed and are becoming increasingly popular. In this context, we aimed at assessing outcomes after HH repair with this new generation of mesh at our institution.

**Methods:**

From a prospective database, we identified all consecutive patients that underwent HH repair with BSM augmentation. Data was extracted from electronic patient charts of our hospital information system. Endpoints of this analysis included perioperative morbidity, functional results and recurrence rates at follow-up.

**Results:**

Between December 2017 and July 2022, 97 patients (elective primary cases *n* = 76, redo cases *n* = 13, emergency cases *n* = 8) underwent HH with BSM augmentation. Indications in elective and emergency cases were paraesophageal (Type II–IV) HH in 83%, and large Type I HH in 4%. There was no perioperative mortality, and overall (Clavien–Dindo ≥ 2) and severe (Clavien–Dindo ≥ 3b) postoperative morbidity was 15% and 3%, respectively. An outcome without postoperative complications was achieved in 85% of cases (elective primary surgery 88%, redo cases 100%, emergencies cases 25%). After a median (IQR) postoperative follow-up of 12 months, 69 patients (74%) were asymptomatic, 15 (16%) reported improvement, and 9 (10%) had clinical failure, of which 2 patients (2%) required revisional surgery.

**Conclusion:**

Our data suggest that HH repair with BSM augmentation is feasible and safe with low perioperative morbidity and acceptable postoperative failure rates at early to mid-term follow-up. BSM may be a useful alternative to non-resorbable materials in HH surgery.

The problem of herniation after sutured closure of human fascia is as old as modern surgery. In the 1870s, it was Theodor Billroth who stated: “If we could artificially produce tissue of the density and toughness of fascia and tendon, the secret of radical hernia cure would be discovered”. In 1957, the discovery to synthesize polypropylene at an industrial scale [1] was the stepping stone for the success of surgical mesh reinforcement, which has become the uncontested standard of care for incisional and inguinal hernia, rectal and vaginal prolapse, and many other hernia types [2–6].

However, mesh reinforcement in hiatus hernia (HH) surgery has always been controversial, and many surgeons avoid crural prostheses because of the lack of convincing scientific evidence, the risk of severe complications, and the technical challenges of revisional surgery [7]. On the other hand, mesh supporters argue that augmentation should be performed particularly in large HH to reduce the high risk of long-term recurrence and, most importantly, to avoid complex reoperations [8]. In addition, mesh-related morbidity such as stenosis and erosion appears to be rare with a reported incidence of 0.035% [9], and severe complications occur almost exclusively after reinforcement with non-absorbable synthetic materials [10].

In an attempt to overcome the undesirable characteristics of permanent synthetic meshes, absorbable allogenic and xenogeneic materials (“biomeshes”) have been introduced and widely promoted. Biomesh is rapidly revascularized and has a high resistance to bacterial contamination [11, 12]. However, high cost, negative long-term results in randomized trials [13], and cultural and religious issues [14] have prevented widespread use of these materials.

New-generation long-term absorbable biosynthetic meshes (BSM) have recently been developed to combine the advantages and avoid the downsides of synthetic materials and biomeshes. Phasix ST® (BD, Allschwil, Switzerland) is made from poly-4-hydroxybutyrate (P4HB), a material that handles well laparoscopically, absorbs and remodels to native host tissue within 6–18 months [15, 16], and should therefore carry a lower risk of long-term complications. Although promising in concept, only few studies have reported clinical outcomes after P4HB reinforcement in HH repair [17–19]. Hence, the aim of this study was to analyze our institutional experience with this new generation of mesh.

## Methods

### Study design

From a prospective database, we identified all consecutive patients that underwent HH repair with BSM augmentation at our institution. Data relevant to this study such as basic patient demographics, pre- and postoperative clinical work-up, and details of surgical therapy were gathered from both our prospective database and the electronic patient charts of our hospital information system. The study was approved by the Ethical Committee of the Canton of Zurich (BASEC Nr.: 2021-00822).

### Preoperative work-up and surgical technique

Routine preoperative work-up included endoscopy and radiography (contrast swallow or CT-scan). In addition, functional investigations (esophageal pH monitoring and manometry) were performed in patients with esophagitis, reflux symptoms, and dysphagia. In contrast, esophageal pH monitoring and manometry were facultative in large Type III or Type IV HH, particularly in cases with chronic anemia and Cameron lesions.

**Fig. 1:**
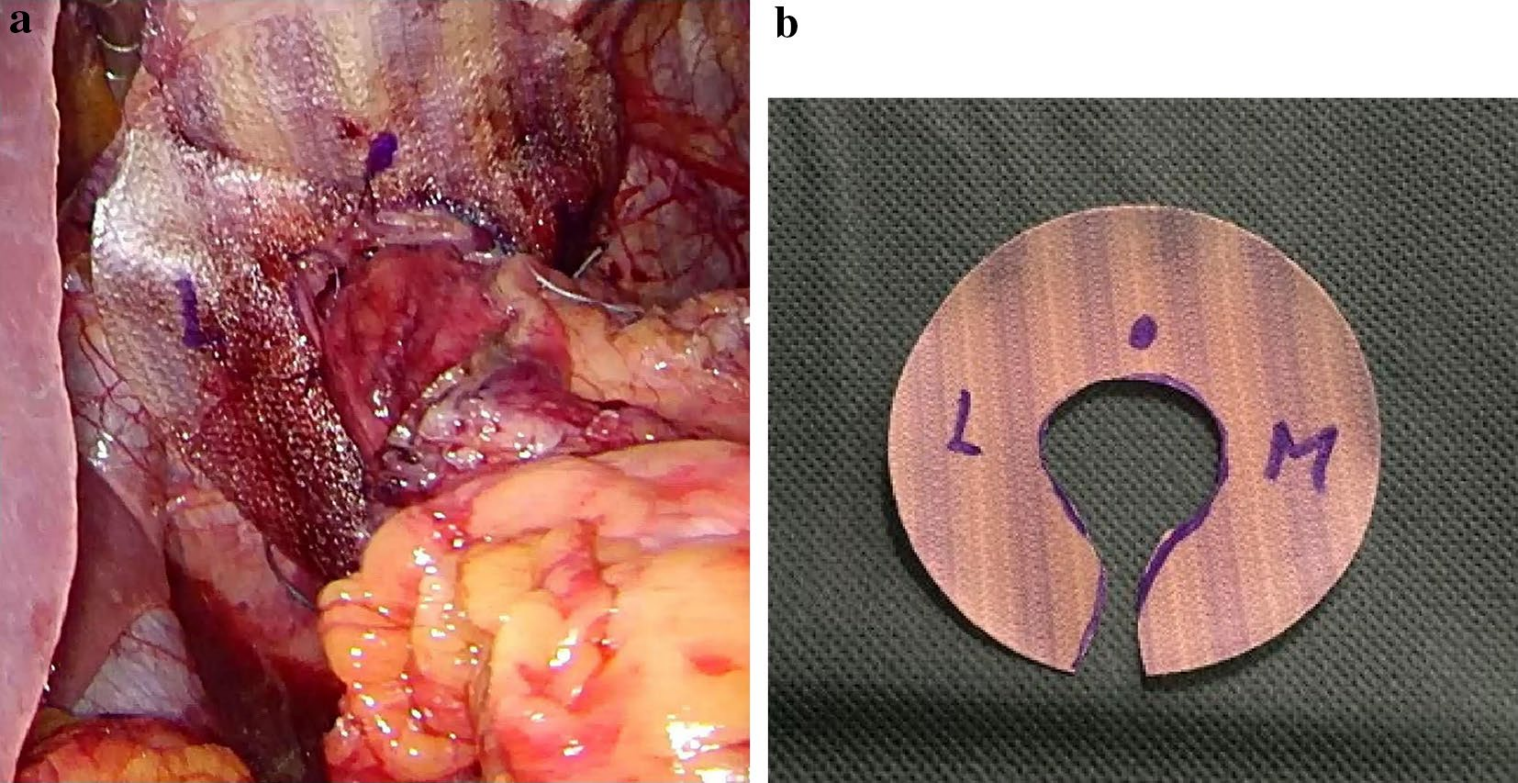
**a** Intraoperative aspect of the esophageal hiatus reinforced with circular Phasix ST™ (BD) patch. **b** Aspect of the circular Phasix ST™ (BD) patch. Note that the central recess is placed 1-2 cm below the center of the patch to achieve a wider covering of the anterior hiatus. Markings to guide mesh positioning in German language *L* Leber (liver) and *M* Milz (spleen)

All surgical procedures were performed in a modified “French position” with 20–25° anti-Trendelenburg tilt and the surgeon standing between the patient’s legs. Five trocars were used for the laparoscopic approach. Dissection of the esophageal hiatus usually started at the right crus after division of the lesser omentum. After identification of the phreno-esophageal membrane and dissection of the hernia sac (if present), mediastinal mobilization was performed until the intraabdominal esophageal segment reached a sufficient length of 2–3 cm without longitudinal traction. Reconstruction of the hiatus was then performed in all patients with a posterior crurorrhaphy using 3–4 single form-8 stitches (Ethibond 0, Ethicon, Zug, Switzerland) and—in large hiatus defects—an additional left anterior cruroplasty with the same suture material. One patient underwent an additional Collis procedure for esophageal lengthening. No diaphragmatic relaxing incisions were performed. For mesh augmentation, a circular (8 cm diameter) monofilament P4HB patch with a hydrogel barrier on the abdominal surface (Phasix™ ST Mesh, BD) was used. Prior to implantation, the patch was modified with a 2.5–3 cm central recess for the esophagus via a radial incision. The central recess was placed 1–2 cm caudally from the center of the patch to create a wider cover on the anterior hiatal circumference, which was considered the weakest point of the reconstruction. The radial incision to create the recess was placed posteriorly to provide uninterrupted anterior cover of the hiatus (Fig. 1a). After placement around the abdominal esophagus in an onlay fashion, the mesh was fixed at the hiatus with 4–6 absorbable sutures (Vicryl 3-0, Ethicon). Additional sphincter augmentation with total or partial fundoplication, magnetic sphincter augmentation, fundo-phrenicopexy, His-angle reconstruction (Lortat-Jacob), or Hill gastropexy was performed according to the patients’ individual pathophysiology, symptom profile, and surgeons’ preference.

### Postoperative follow-up and outcome measures

All patients were offered routine functional aftercare including standardized assessment of symptoms and contrast UGI radiography. In addition, patients with esophagitis or Barrett’s esophagus were routinely followed up endoscopically. Outpatient visits were scheduled at 6 weeks, 3 months and then annually. All patients who missed routine follow-up visits were contacted and invited for an individual appointment at our outpatient clinic. Outcome measures included postoperative morbidity, intensive care unit (ICU) and hospital stay, hospital readmission, and failure rates (clinical, radiological, and endoscopic). Postoperative complications were graded according to the Clavien–Dindo (CD) classification [20] and the Comprehensive Complication Index (CCI) at 30 and 90 days after surgery [21]. Recurrence was defined as any size hernia identified on postoperative UGI radiography or endoscopy.

### Statistical analysis

Numerical variables were summarized using medians and interquartile ranges (IQR). Categorical variables were summarized using counts and percentages. The *t*- or Wilcoxon tests (continuous variables) or chi-square/Fisher tests (proportions) were used as appropriate. Linear mixed-effects models, which account for correlated (within patient) data, was used to evaluate changes in patients’ QoL over time. Overall recurrence rates were projected using Kaplan–Meier estimation and 95% confidence intervals. For all testing, a *p* < 0.05 was considered statistically significant. All statistical analysis was performed with IBSM SPSS software (version 24.0, SPSS Inc, Armonk, NY, USA).

## Results

### Patient characteristics and surgical procedures

Between December 2017 and July 2022, 97 patients underwent HH repair with BSM reinforcement at our department. Basic patient demographics, preoperative symptom profiles, and details of the preoperative work-up are summarized in Table 1. The predominant indication for surgery was a primary Type III hiatus hernia (77%). Emergencies and recurrent hernia accounted for 8% and 13% of cases, respectively. Redo cases had a history of one or two previous HH repairs in 11% and 3%, respectively.

**Table 1:** Patient characteristics, symptoms and preoperative work-up

	Total (*n* = 97)
Basic demographics	
Male, *n* (%)	38 (39%)
Age, years, median (IQR)	65 (18.6)
Body mass index, kg/m^2^, median (IQR)	27 (6)
ASA score, median (IQR)	3 (1)
Overall symptom duration, months, median (IQR)	17 (54.8)
Preoperative symptom profile, *n* (%)	
Heartburn	74 (76%)
Chest pain	50 (52%)
Regurgitation	33 (34%)
Dysphagia	32 (33%)
Dyspnoea	29 (30%)
Fullness/Bloating	25 (26%)
Nausea	21 (22%)
Chronic anemia with previous transfusions	21 (22%)
Unintended weight loss	16 (16%)
Cardiac arrhythmia	3 (3%)
Preoperative workup, *n* (%)	
UGI endoscopy	93 (96%)
Computed tomography	56 (58%)
High-resolution manometry	46 (46%)
Contrast radiography	42 (43%)
24 h-pH study	41 (42%)
Hiatal hernia types (Skinner classification), *n* (%)	
Type I	4 (4%)
Type II	0 (0%)
Type III	75 (77%)
Type IV	5 (5%)
Recurrent hernia	13 (13%)
First recurrence	10 (11%)
Second recurrence	3 (3%)

*IQR* interquartile range, *UGI* upper gastrointestinal

Most patients underwent laparoscopic access surgery (92%) in an elective setting (92%). In all cases, a circular Phasix™ ST (BD) mesh was placed around the esophagus in a circular fashion and fixed with absorbable sutures. Details of surgical interventions including all concomitant procedures are displayed in Table 2.

**Table 2:** Surgical procedures

	Total (*n* = 97)	Elective primary surgery (*n* = 76)	Revisional surgery (*n* = 13)	Emergency surgery (*n* = 8)
General, *n* (%)				
Duration of surgery (skin-to-skin), minutes, median (IQR)	178 (86.5)	170 (67)	205 (77)	221 (124)
Laparoscopic approach	89 (92%)	71 (93%)	11 (85%)	7 (88%)
Open approach	4 (4%)	1 (1%)	2 (15%)	1 (12%)
Robotic approach	4 (4%)	4 (5%)	0 (0%)	0 (0%)
Conversion (laparoscopic to open)	2 (2%)	1 (2%)	1 (8%)	0 (0%)
Crural repair, *n* (%)				
Anterior and posterior	80 (84%)	64 (84%)	8 (62%)	8 (100%)
Posterior only	14 (14%)	11 (14%)	3 (23%)	0 (0%)
Anterior only	3 (3%)	1 (1%)	2 (15%)	0 (0%)
Concomitant surgical procedures, *n* (%)				
Related				
Gastropexy/Fundo-phrenico-pexy	72 (74%)	55 (72%)	9 (69%)	8 (100%)
Nissen fundoplication	21 (22%)	17 (22%)	4 (31%)	0 (0%)
Magnetic sphincter augmentation	9 (9%)	7 (9%)	2 (15%)	0 (0%)
Posterior (Toupet) fundoplication	5 (5%)	3 (6%)	2 (15%)	0 (0%)
Collis gastroplasty	1 (1%)	1 (1%)	0 (0%)	0 (0%)
Anterior (Dor/Watson) fundoplication	1 (1%)	1 (1%)	0 (0%)	0 (0%)
Roux-en-Y gastric bypass	1 (1%)	0 (0%)	1 (8%)	0 (0%)
Unrelated				
Adhesiolysis	8 (8%)	2 (3%)	6 (46%)	0 (0%)
Ventral hernia repair	4 (4%)	3 (4%)	1 (8%)	0 (0%)
Bowel resection	2 (2%)	0 (0%)	1 (8%)	1 (12%)
Diaphragmatic hernia (Morgagni and Larrey)	1 (1%)	1 (1%)	0 (0%)	0 (0%)
Liver resection	1 (1%)	1 (1%)	0 (0%)	0 (0%)
Hartmann’s procedure	1 (1%)	1 (1%)	0 (0%)	0 (0%)

### Perioperative outcomes

The median ICU and hospital stay was 0 and 4 days, respectively. Only emergency cases required ICU care. All intra- and postoperative complications are listed in Table 3. An outcome without postoperative complications in elective primary, redo, and emergencies cases was achieved in 88%, 100%, and 25%, respectively. Mortality was 0% within 30 days after surgery, and overall (CD ≥ 2) and severe (CD ≥ 3b) morbidity was 15% and 3%, respectively. Perioperative morbidity was significantly (*p* < 0.05) higher in emergency cases. No intraoperative complications and no intra- and postoperative mesh-related morbidity was encountered.

**Table 3:** Perioperative outcomes

	Total (*n* = 97)	Elective primary surgery (*n* = 76)	Revisional surgery (*n* = 13)	Emergency surgery (*n* = 8)
Postoperative complications, *n* (%)				
Pulmonary	6 (6%)	2 (3%)	0 (0%)	4 (50%)
Intestinal paralysis	5 (5%)	4 (5%)	0 (0%)	1 (13%)
Wound infection	4 (4%)	3 (3%)	0 (0%)	1 (13%)
Cardiac	3 (3%)	2 (3%)	0 (0%)	1 (13%)
Intra-abdominal abscess	1 (1%)	0 (0%)	0 (0%)	1 (13%)
Fascia dehiscence	1 (%)	1 (1%)	0 (0%)	0 (0%)
Highest Clavien–Dindo grade, *n* (%)				
Grade II	12 (12%)	7 (9%)	0 (0%)	5 (63%)
Grade IIIa	0 (0%)	0 (0%)	0 (0%)	0 (0%)
Grade IIIb	2 (2%)	2 (3%)	0 (0%)	0 (0%)
Grade IVa	0 (0%)	0 (0%)	0 (0%)	0 (0%)
Grade IVb	1 (1%)	0 (0%)	0 (0%)	1 (13%)
Grade V	0 (0%)	0 (0%)	0 (0%)	0 (0%)
Postoperative complications summary				
Textbook outcome, *n* (%)	82 (85%)	67 (88%)	13 (100%)	2 (25%)
ICU stay, days, median (IQR)	0 (0)	0 (0)	0 (0)	0 (0)
Hospital stay, days, median (IQR)	4 (2)	4 (2)	4 (1)	8 (3)
CD ≥ II, *n* (%)	15 (15%)	9 (12%)	0 (0%)	6 (75%)
CD ≥ IIIb, *n* (%)	3 (3%)	2 (3%)	0 (0%)	1 (13%)
CCI at 30 days, median (IQR)	0 (20.9)	0 (8.7)	0 (8.7)	21 (13)
CCI at 90 days, median (IQR)	0 (14.8)	0 (6.5)	4 (8.7)	21 (12)
Hospital readmission within 90 days, *n* (%)	6 (6%)	5 (7%)	1 (8%)	0 (0%)

*CD* Clavien–Dindo grade, *CCI* comprehensive complication index; Textbook outcome = no perioperative complications CD ≥ II

### Outcomes at follow-up

The median (IQR) follow-up was 12 [17] months, 93 patients (96%) were followed up regularly, and 4 patients (4%) were lost to follow up. At follow-up, complete success (defined as being asymptomatic without evidence of anatomical HH recurrence) was achieved in 69 patients (74%), partial success (defined as symptom improvement with or without anatomical HH recurrence or asymptomatic anatomical HH recurrence) was achieved in 15 patients (16%), and 9 patients (10%) reported clinical failure (defined as unchanged or worsened symptoms with or without anatomical recurrence). A detailed symptom analysis in patients with clinical failure is outlined in Table 4.

**Table 4:** Outcomes at follow-up

	Total (*n* = 97)	Elective primary surgery (*n* = 76)	Revisional surgery (*n* = 13)	Emergency surgery (*n* = 8)
Duration of FU, months [median (IQR)]	12 (17)	12 (15)	17 (13)	11 (19)
Lost to FU, *n* (%)	4 (4%)	3 (4%)	0 (0%)	1 (12.5%)
Clinical and objective assessment at FU, *n* (%)				
Clinical evaluation	93 (100%)	73 (100%)	13 (100%)	7 (100%)
Contrast radiography	55 (59%)	40 (55%)	10 (77%)	5 (71%)
Endoscopy	25 (27%)	16 (22%)	8 (62%)	1 (14%)
Symptoms and HH recurrence at FU, *n* (%)				
Complete success	69 (74%)	59 (81%)	4 (31%)	6 (86%)
Partial success	15 (16%)	8 (11%)	6 (46%)	1 (14%)
Clinical failure	9 (10%)	6 (8%)	3 (23%)	0 (0%)
Anatomical failure	8 (9%)	4 (5%)	3 (23%)	1 (14%)
Symptoms in patients with clinical failure, *n* (%)				
Reflux	7 (70%)	4 (67%)	3 (100%)	0 (0%)
Dysphagia	5 (50%)	3 (50%)	2 (67%)	0 (0%)
Postprandial heartburn/chest pain	5 (50%)	2 (33%)	3 (100%)	0 (0%)
Fullness/Bloating	3 (30%)	2 (33%)	1 (33%)	0 (0%)
Regurgitation	1 (10%)	1 (17%)	0 (0%)	0 (0%)
Nausea	1 (10%)	0 (0%)	1 (33%)	0 (0%)
Pulmonary symptoms	1 (10%)	0 (0%)	1 (33%)	0 (0%)

*FU* follow-up, *HH* hiatus hernia, Complete success: asymptomatic, no objective evidence of HH recurrence; Partial success: symptom improvement ± objective evidence of HH recurrence or asymptomatic + objective evidence of HH recurrence; Clinical failure: Unchanged or worsened symptoms ± objective evidence of HH recurrence; Anatomical failure: objective evidence of HH recurrence ± symptoms

Upper gastrointestinal endoscopy and/or contrast swallow studies were performed in 25 (27%) and 55 patients (59%) at follow-up, respectively. Anatomical HH recurrence was evidenced in 8 patients (9%). Two patients required surgical revision for symptomatic hernia recurrence after 23 and 34 months, respectively. At reoperation, complete absorption of the mesh with mild adhesions to adjacent organs was noted.

## Discussion

The present study is currently the largest reporting on BSM augmentation in HH repair and adds further evidence to the existing literature. Similar to earlier series using P4HB patches [17–19], we observed no severe mesh-related intra- and perioperative morbidity, and clinical results after mid- and long-term follow-up were encouraging with good functional results, a low objective recurrence rate and no evidence of erosions or stenosis. In agreement with the previously published experience, we may therefore conclude that BSM with P4HB patches can be safely performed at the hiatus. [2–7, 9, 22].

Although—from a physical point of view—it may seem obvious that meshes increase the tensile strength of the reconstructed hiatus, the general indication for mesh augmentation in HH surgery remains a hotly contested topic. In this regard, the current evidence from randomized controlled trials (RCT) [8, 13, 23–28] and meta-analyses [29–33] shows no clear advantage of mesh augmentation compared with sutured closure alone during short- and long-term follow-up. Nevertheless, the available evidence is difficult to interpret owing to different indications, mesh materials, surgical techniques, definitions of recurrence, and durations of follow-up. Indeed, mesh augmentation at the hiatus is far from standardized and there are various materials (synthetic, biosynthetic, biological, absorbable, and non-absorbable) with diverging characteristics on the market. Meshes come in many different sizes and shapes (rectangular, circular, u-shaped) and can be placed at the hiatus in keyhole, posterior, or anterior fashion or on separate (relaxing) diaphragmatic incisions. Therefore, we may assume that the conflicting evidence regarding mesh augmentation at the hiatus in general is at least in part caused by the technical heterogeneity of the surgical approaches [29, 34].

In this context, it may be revealing to review the relationship between anatomical patterns of HH recurrence and the duration of follow-up. From several well-performed retrospective cohort studies we know that most early recurrences (< 12 months) [35, 36], are typically located posteriorly or circumferentially in consequence of a disrupted crurorrhaphy and may therefore be considered a technical failure; i.e. a true recurrence [37]. In contrast, recurrent HH after long-term follow-up (> 12 months) seems to have a different pathophysiology as it is mostly found at the anterior hiatus [37–39] as a result of the constant physiological strain leading to stretch and widening of the weakest part of the hiatus over time. We agree with others [40] that early and late HH recurrence should therefore be seen as separate entities, and we would like to stress that this distinction must be considered when analyzing the current literature.

In this context, there is growing evidence from the literature that circular mesh placement leads to fewer recurrences compared with u-shaped posterior mesh configuration [41]. On the other hand, “keyhole” placement of non-absorbable materials is avoided by many surgeons because of the risk of mesh shrinkage and stenosis at follow-up. Therefore, mesh augmentation has been performed posterior-only in most published RCTs without adequate reinforcement of the anterior “weak spot” of the hiatus. As a result, long-term HH recurrence rates were similar or even higher after mesh augmentation in the “posterior-only” RCTs [13, 26–28, 42], but significantly lower in RCTs employing circular [23] or combined posterior and anterior mesh reinforcement [25]. We may therefore speculate that insufficient anterior augmentation in most included RCTs may have contributed to the negative results of a recent meta-analysis [29].

With this in mind, and considering the fact that up to 75% of recurrences are located anteriorly, we have changed our institutional technique of mesh configuration by placing the recess for the abdominal esophagus 1–2 cm below the center of the patch to achieve an even wider coverage of the anterior hiatus (Fig. 1b).

There are certain limitations associated with our study. Most importantly, as this is a prospective cohort study of consecutive cases undergoing BSM augmentation, we were not able to compare patients to a “suture only” control group. Thus, an adequately powered registry analysis or preferably an RCT with clearly defined and standardized indications, surgical procedures and quality assurance would be the next step in evaluating the therapeutic strategies for HH repair.

In conclusion, this study confirms that reinforcement of crurorrhaphy with the Phasix ST® (BD) P4HB patch is feasible and clinically effective in the short- and mid-term follow-up. Furthermore, our series provides additional evidence that the safety profile of long-term absorbable BSM is excellent with a very low rate of mesh-related complications even in a “keyhole” position encircling the abdominal esophagus. Nevertheless, the ideal technique for hiatus reconstruction has yet to be determined and—particularly with regard to the rapidly rising incidence of large HH in Western societies [43]—well-designed prospective and preferably randomized studies are needed to confirm long-term reliability of new-generation long-term absorbable BSM.
